# Biological characterization of a novel in vitro cell irradiator

**DOI:** 10.1371/journal.pone.0189494

**Published:** 2017-12-12

**Authors:** Tyler L. Fowler, Michael M. Fisher, Alison M. Bailey, Bryan P. Bednarz, Randall J. Kimple

**Affiliations:** 1 Department of Medical Physics, University of Wisconsin School of Medicine and Public Health, University of Wisconsin, Madison, WI, United States of America; 2 Department of Human Oncology, University of Wisconsin School of Medicine and Public Health, University of Wisconsin, Madison, WI, United States of America; 3 University of Wisconsin Carbone Comprehensive Cancer Center, University of Wisconsin School of Medicine and Public Health, University of Wisconsin, Madison, WI, United States of America; Tulane University Health Sciences Center, UNITED STATES

## Abstract

To evaluate the overall robustness of a novel cellular irradiator we performed a series of well-characterized, dose-responsive assays to assess the consequences of DNA damage. We used a previously described novel irradiation system and a traditional ^137^Cs source to irradiate a cell line. The generation of reactive oxygen species was assessed using chloromethyl-H_2_DCFDA dye, the induction of DNA DSBs was observed using the comet assay, and the initiation of DNA break repair was assessed through γH2AX image cytometry. A high correlation between physical absorbed dose and biologic dose was seen for the production of intracellular reactive oxygen species, physical DNA double strand breaks, and modulation of the cellular double stand break pathway. The results compared favorably to irradiation with a traditional ^137^Cs source. The rapid, straightforward tests described form a reasonable approach for biologic characterization of novel irradiators. These additional testing metrics go beyond standard physics testing such as Monte Carlo simulation and thermo-luminescent dosimeter evaluation to confirm that a novel irradiator can produce the desired dose effects *in vitro*. Further, assessment of these biological metrics confirms that the physical handling of the cells during the irradiation process results in biologic effects that scale appropriately with dose.

## Introduction

The study of the radiosensitivity of cells in culture has been an important component of radiobiology research for nearly a century [1]. Many investigators continue to utilize self-shielded radioactive sources utilizing ^137^Cs that currently necessitate background checks and security clearance to access, in addition to requiring significant overhead to maintain [2]. These irradiators often require careful placement of samples within the housing to avoid inhomogeneous dosing and, depending on the design, can suffer from dose gradients due to shutter lag. Due to the >30 year half-life of ^137^Cs, these systems also provide a highly stable radiation dose rate over the time course of a typical experiment. Due to increasing concerns about the management and security of radioactive materials in research environments, there has been a gradual and likely permanent shift to machine-based x-ray irradiators [2].

X-ray irradiators have become a popular alternative to ^137^Cs irradiators due to a number of factors including lower cost, smaller physical footprint and easier maintenance. Conventional x-ray irradiators used to investigate biological responses are built to provide maximum versatility to radiobiology researchers by accommodating small animals, tissue samples, and cellular applications. The tradeoff for these advantages include the potential for a “heel effect”, ramp up/down dose delivery, and output drift over days to weeks. These effects can result in variation in radiation dose delivery depending upon the system and highlight the need for both physical and biologic characterization of novel irradiators.

Over the last several years, we have developed a micro-irradiator capable of delivering multiple dose and dose-rates within a single cell-culture microplate for use in high-throughput assays. We have previously described the physical characteristics of this system [3]. The design of any high throughput technology for *in vitro* biology research must limit the impact of mechanical and environmental stresses on the cells being tested. To evaluate the overall robustness of this novel irradiator with regard to the effects of the irradiator workflow on biologic endpoints, we performed biological characterization using several assays focused on the early biologic events seen following a dose of ionizing radiation: (1) the production of inter-cellular reactive oxygen species, (2) the production of physical DNA double strand breaks, and (3) early signals of the DNA double-strand break repair pathway.

## Results

**Fig 1:**
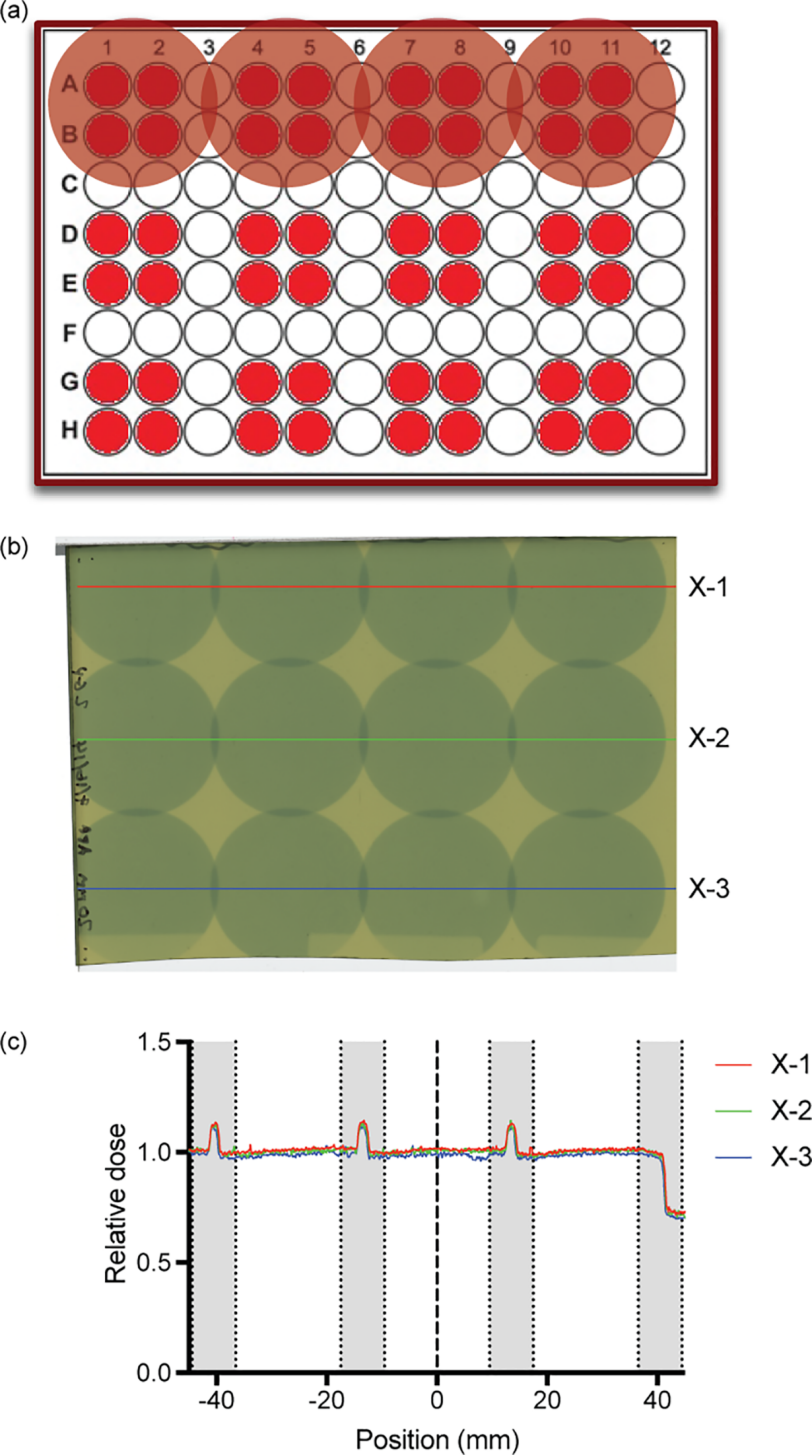
Irradiation schematic and dose verification. (a) Schematic of four well irradiation method. (b) GafChromic EBT-3 film irradiated with 20 mm applicator to a dose of 2 Gy/field. Labeled lines represent the density used to graph (c) Calculated relative density of labeled lines. Non-target regions are shaded grey.

We have previously described the detailed physical dosimetry and specifications of our irradiator [3]. We used our previously described 96 well plate phantom to assess the contribution of scatter radiation when using the 20-mm surface applicator. Commonly used radiation doses for biologic studies are in the range of 2–8 Gy [4–6]. A 2 Gy dose was delivered and the delivered dose was normalized to the center of the 4 target wells. By leaving a single space well between target wells (Fig 1A), scatter dose had negligible effect on target wells (Fig 1B and 1C).

To confirm that this system was capable of delivering biologically relevant radiation doses, we devised a three-step process for biologic characterization investigating early effects of radiation delivery. Due to production of physiologic ROS, and repair of DNA strand breaks over time, these approaches are not appropriate for absolute dosimetry, but rather provide confirmation that anticipated biological effects are seen.

**Fig 2:**
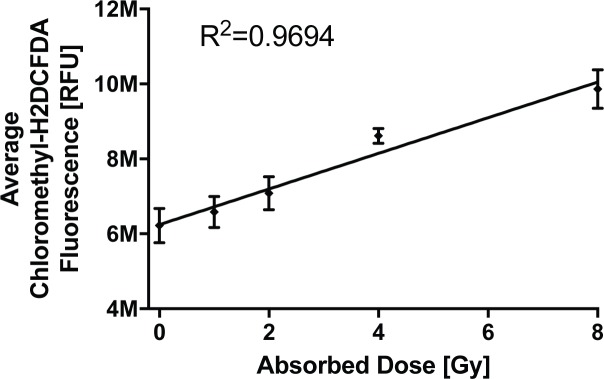
Reactive oxygen species production by radiation. Production of reactive oxygen species by ionizing radiation scaling linearly with increasing absorbed dose to the cells (+/- 95% CI). Show are results from one of 3 representative experiments with 12 replicate wells per dose.

The generation of reactive oxygen species due to photons interacting with molecules inside the cell (e.g. H_2_O) is one of the earliest initial effects of ionizing radiation. We assessed the relative change in production of intracellular ROS using a fluorescent assay well suited to high-throughput screening. With increasing radiation dose, a significant increase in ROS as measured by chloromethyl-H_2_DCFDA fluorescence was seen (Fig 2). This increase scaled linearly with radiation dose over the range of 0–8 Gy (R^2^ = 0.9229).

**Fig 3:**
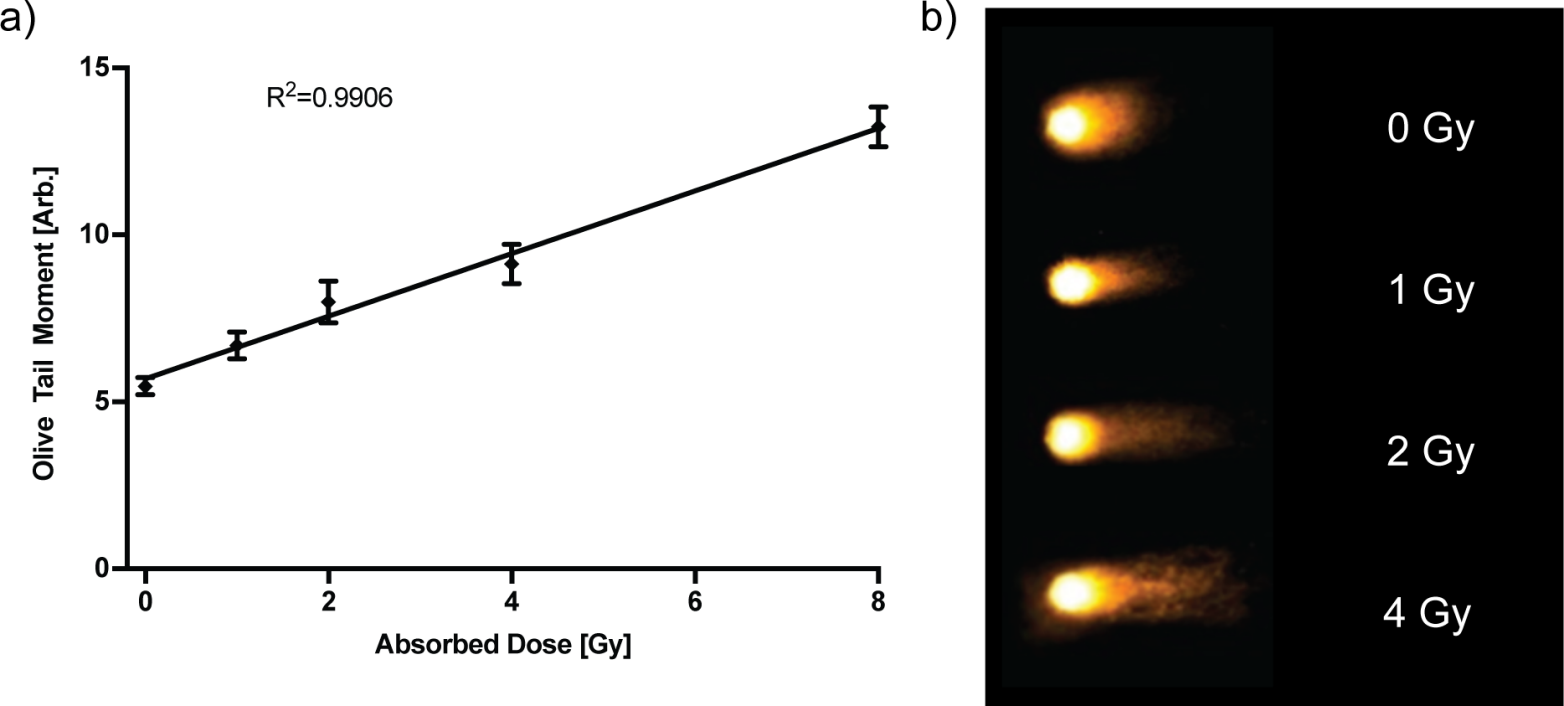
Double strand breaks generated by radiation. (a) Production of physical DNA double strand breaks by ionizing radiation scaling linearly with increasing absorbed dose to the cells (+/- 95% CI). Show are pooled results from 3 replicate experiments. (b) Representative comets at several dose points at 20X stained with SYBR Green.

Next, DNA damage from both ROS production near DNA and direct ionization can be evaluated through measurement of physical DNA double strand breaks (DSB). DSBs scale linearly with ionizing radiation dose, with an incidence of approximately 20–40 DSBs per Gy of absorbed dose per nucleus from x-rays [7, 8]. The measurement of physical DNA DSBs created by ionizing radiation was achieved by performing single cell gel electrophoresis (a.k.a "neutral comet assay") where the DNA fragments are pulled out of the cell. Smaller DNA fragments produced by irradiation migrate further through the agarose gel forming a longer comet tail. We used CaspLab automatation software to quantify the Olive Tail Moment (OTM) [9], a measure of DNA damage [10]. We again irradiated cells with a range of radiation doses across a single 96 well plate. In this manner, a large number of comets were analyzed per dose point (Table 1). The average OTM of the samples increased linearly with radiation dose (Fig 3, R^2^ = 0.9906).

**Table 1:** Number of comets assayed over 3 replicate experiments.

Absorbed Dose [Gy]	# Comets Counted
0	434
1	372
2	177
4	194
8	273

**Fig 4:**
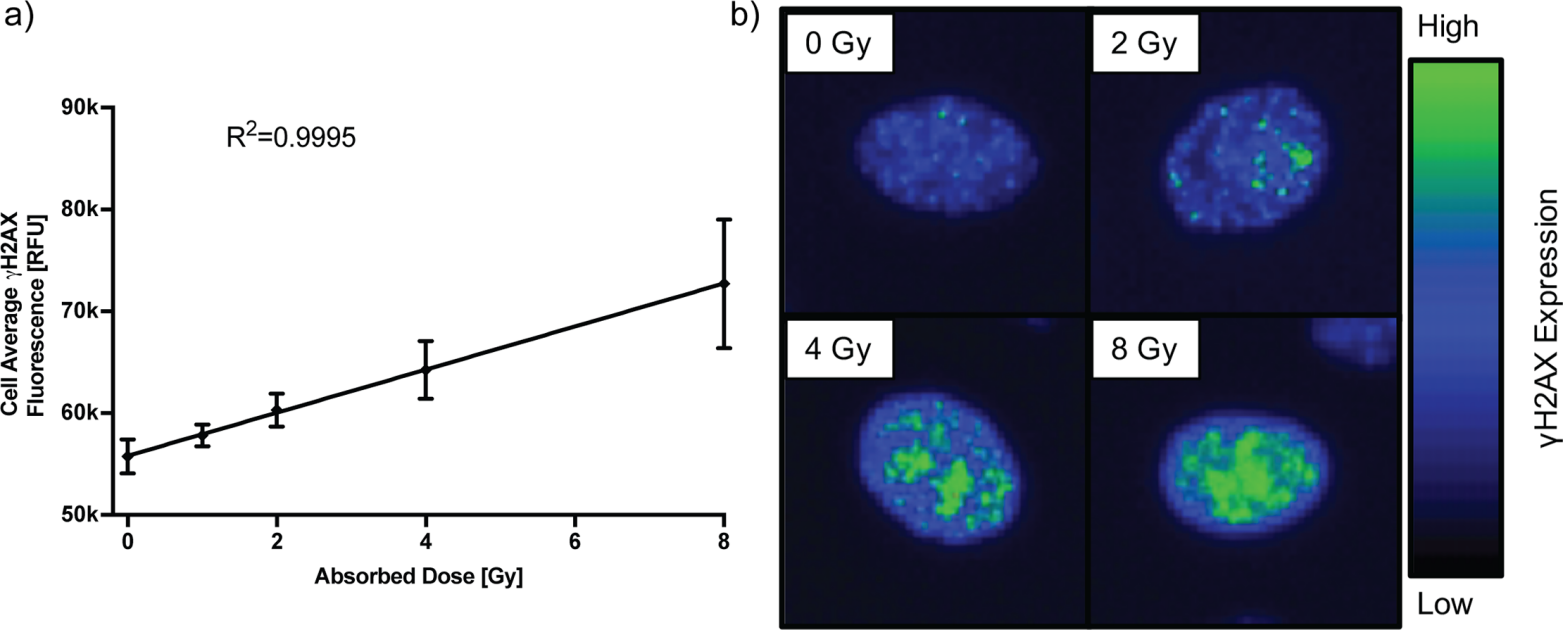
Marker of DNA repair. (a) Phosphorylation of histone 2AX, activating the DNA double strand break repair pathways, by ionizing radiation scaling linearly with increasing absorbed dose to the cells (+/- 95% CI). Shown are results from one of 3 representative experiments with 8 replicate wells per dose. (b) Representative IF microscopy images of γH2AX foci at several dose points at 40X magnification.

Finally, following the induction of DNA strand breaks, the DNA repair machinery identifies the break sites and activates a complex sequence of events with the goal of repairing the induced DNA damage. One of the early steps in this process is phosphorylation of the histone H2AX at serine 139 (i.e. γH2AX), an event that can be identified using immunofluorescence. We utilized a microplate assay of γH2AX fluorescence which has high correlation with approaches that count individual foci[11]. The cell average γH2AX fluorescence increases linearly with increasing absorbed dose (Fig 4, R^2^ = 0.9995).

To confirm that these assays would provide linear responses in cells treated with a ^137^Cs irradiator, all experiments were also performed using our JL Shepard irradiator. Due to the inability of the Cs irradiator to deliver a different dose to individual wells of a single plate our experimental setup was altered such that each dose was delivered to an individual plate. This resulted in greater dose-response heterogeneity (i.e. lower R^2^ values), but a similar dose response was seen for each assay (S1 Fig).

## Discussion

There is a critical need to enhance pre-clinical in vitro testing of novel systemic agents in combination with radiation to improve outcomes for patients with hard to treat cancers. While most work over the last several decades has been performed using ^137^Cs systems, future studies will likely be performed using a variety of novel irradiators, some commercial systems and some “home-grown”. Herein we present a biological method for final characterization of a novel irradiator system by analyzing a cascade of three distinct events associated with DNA damage that are known to be linearly related to absorbed dose. Using these assays, we have shown negligible perturbation being introduced by our system during cellular irradiation and increased the confidence in the use of our system.

Modern cancer biology research often utilizes high-throughput assays performed in multi-well plates (e.g. 96- or 384-wells per plate) that can be rapidly analyzed using high sensitivity endpoint assays. Experiments with multiwall plates are designed such that an entire experiment is included on a single plate as between plate comparisons can increase heterogeneity due to differences in timing, subtle changes in plate chemistry, and read-to-read variability. Current x-ray or radioactive source-based irradiators are incapable of varying the dose and/or dose-rate across the multiple wells in an individual microplate. This necessitates the comparison across multiple plates for investigators interested in either dose or dose/rate effects. Given the small volume of the wells in multi-well plates, small differences in the volume of media overlying the cells can also result in up to a 20% differences in the dose received for kV energy “top-down” irradiators [3]. Fulkerson and colleagues have previously described a steep dose falloff in the first 3–5 mm depth in water using the Xoft Axxent source [12]. The location of a plate within the radiation field can also influence homogeneity across a plate due to the “heel effect” leading to subtle biases in assay interpretation. Important dosimetry and calibration characteristics (i.e. dose build-up region, beam attenuation, and beam scatter) of these irradiators may be unknown to the end user. This gap in irradiator specificity and user knowledge can result in significant deviation between delivered dose and intended dose that ultimately adversely impacts experimental results and reproducibility.

The production of this irradiator system leverages the increased productivity and capability offered by the newest generation of instrumentation used in the radiobiology laboratory. The simple experiments performed herein can form a framework for simple characterization of novel irradiators to ensure that delivery of radiation results in both expected physical dose delivery but also anticipated biologic effects. Novel in vitro radiation systems have the potential to accelerate our understanding of the biological mechanisms that influence radiation sensitivities and may pave the way towards truly delivering personalized medicine in the radiation oncology clinic.

While several novel irradiation devices have been developed in the last decade these mainly focus on producing thin micro-beams that measure several hundred microns in width and require custom fabricated cellular growth plates that are incompatible with standard bioassay instrumentation [13, 14]. Our system was designed from the beginning with the radiobiology researcher in mind to deliver highly uniform, full-well dose to standard cell culture plates. Furthermore, our system incorporates an on-board ionization chamber for full NIST traceable verification of dosimetry prior to an irradiation cycle capable of both delivering multiple doses and dose-rates within the same cell culture plate while traditional Cs-137 and cabinet x-ray irradiators are only capable of delivering single dose and dose-rates to an entire plate [3]. While the current system design could require up to an hour to deliver radiation to an entire plate, it would be possible to construct a similar system with multiple independently controllable sources to drop the irradiation time by up to an order of magnitude. We are investigating the use of a variety of approaches to keep the temperature of cells constant throughout the period of irradiation.

To ensure accurate and precise bioassay results, the design of any high throughput technology for *in vitro* biology research must limit the impact of mechanical and environmental stresses on the cells being tested. The responses of radiation-related assays are critically dependent on timing from delivery of radiation. To address this issue, we carefully timed the completion of radiation for each group of wells to the assay endpoint for the same group. In this way, we were able to minimize effects due to irradiation time and focus this analysis on the effects of the radiation itself. Therefore, in order to evaluate the overall robustness of an irradiator, it is our opinion that biological characterization should be performed using assays with well-characterized dose-response relationships. In this work, a cascade of three distinct events associated with DNA damage that are known to be linearly related to absorbed dose were examined. A similar biological characterization of a microbeam cellular irradiator using γH2AX dephosphorylation has previously been presented by Bordelon et al. [13]. However, changes in γH2AX can be dependent on multiple cellular factors. This led us to include earlier readouts of radiation dose including generation of ROS and DSBs.

In this work, a cascade of three distinct events associated with the early effects of DNA damage that are known to be linearly related to absorbed dose were examined to characterize the biologic impact and demonstrate the robustness of this high throughput irradiator system [7, 8]. The production of intracellular reactive oxygen species (chloromethyl-H_2_DCFDA dye), the formation of DNA strand breaks (comet assay), and the initiation of DNA repair (γH2AX) all scaled in a highly linear fashion with increasing radiation dose. Excellent correlation was observed between ROS, DNA DSBs, and γH2AX (S2 Fig). We would expect that there will be variation in the quantity of ROS, the number of DNA DSBs, and the intensity γH2AX response between cell lines due to differences in the individual lines. The results presented here suggest that these biologic readouts will vary based on the radiation dose and not the physical system characteristics. Clearly additional work would be required prior to use of this system for irradiation of small animals, cells grown in suspension, or other biologic systems. Use of TLDs or film dosimetry, as we have previously reported for this approach [3], would be necessary prior to utilization.

By evaluating intracellular reactive oxygen species production, physical DNA double strand breaks, and phosphorylation of histone H2AX we have shown negligible perturbation being introduced by our system during the irradiation cycle. These additional testing metrics provide confidence that not only can the dosimetry be confirmed through standard physics testing such as Monte Carlo simulation and thermo-luminescent dosimeter evaluation but also produce the desired dose effects *in vitro*. It is our belief that this is a critical characterization process that should be strongly considered in the commissioning of any biological research irradiator.

## Materials and methods

All assay reagents, antibodies, and protocol recipes are listed in S1 Table. All experiments were carried out in accordance with and under the approval of the University of Wisconsin Office of Biological Safety regulations. The Cs^137^ biological irradiator utilized in this study are licensed by the State of Wisconsin Department of Health Services (WI Lic. No. 025-1323-02) and are owned and operated by UW-Madison. All experiments were repeated at least 3 times on separate days with replicate wells as indicated.

### Cell lines

All experiments in this report were performed using TERT immortalized human tonsillar epithelial (HTE) cells. HTE cells were a kind gift from Dr. Aloysius Klingelhutz (University of Iowa). Cells were cultured in serum free media at 37°C with 5% CO_2_ as we have previously described [15].

### Irradiation

Irradiation of cells with our high-throughput variable dose-rate irradiator was performed as previously described [3]. The system utilizes a 50 kVp source (Xoft, Inc, San Jose, CA, USA) mounted on a custom fabricated motorized stage. The x-ray tube current can be adjusted up to 300 μA using the manufacturing test fixture controller. The commercially available x-ray source is inverted to enable irradiation from the bottom of the plate thus eliminating variability of dose due to the volume of media overlying cells. A standard 20mm circular applicator was used for all assays. A beam profile flattening filter designed specifically for the 20mm circular applicator was used to ensure adequate beam flatness. The reported half-value layer of Aluminum for this applicator and the Xoft source is 1.63mm for HVL1 and 2.45 mm for HVL2[12]. Radiation was delivered at a dose rate of 82 cGy per minute to groups of 4 wells to provide biologic replicates. As we have previously described, a three-axis, motor-driven translation stage was used to achieve precise automation [3]. In this system, the source is fixed to minimize disruption of the coolant and high-voltage supply lines. A 0.2mm gap was used between the surface applicator and translation stage to ensure smooth translation of the stage. The stage was custom designed using the CAD software SolidWorks® (Dassault Systemes SolidWorks Corp., Waltham, MA), fabricated using a 3D acrylic printer, and operated using precision Velmex™ motors (Bloomfield, NY) located on each independent axis. Motors were controlled by LabVIEW (National Instruments Corp., Austin, TX) enabling automated control of the stage position and irradiation dwell time. As we have previously described, the system also includes an Exradin A20 (Standard Imaging, Madison, WI) end-window ionization chamber to provide periodic NIST traceable air-kerma rate verification [3].

In addition to assessing the dose rate at the beginning and end of each radiation delivery using the Exradin A20 ionization chamber, the system was also regularly calibrated by relative film dosimetry using Gafchromic EBT3 film according to the method of McCaw and DeWerd[16]. A custom designed phantom which was machined from a tissue culture plate and incorporated overlying media to provide back-scatter. This system enables us to deliver differing radiation doses to groups of 4 replicate wells. In this way all experiments were performed in 96 well plate format with all radiation doses delivered to a single plate. As demonstrated in our prior publication, we utilize an empty well around each group of 4 wells in a 96 well plate. This “spacer” well ensured that the dose from irradiation of a prior well grouping was less than 1% of the delivered dose.

All radiation was performed at room temperature (22C) although the cells were maintained as close to 37C during transportation through the use of warmed water bottles and an insulated carrier. All cells were kept in a standard incubator following irradiation and until endpoint assays were performed. Irradiation with a traditional ^137^Cs source (JL Shepherd delivering a dose rate of approximately 400 cGy/min) was performed as we have previously described and with similar efforts to maintain sample temperature at 37C throughout each exposure [15]. Due to the inability to modulate the dose delivered to individual wells of a 96 well plate, each radiation dose was delivered to a separate plate. All experiments were performed in triplicate (i.e. on multiple days) and in such a way as to ensure that the time from irradiation to biologic assay was consistent for each radiation dose. For each assay results from a single experiment with replicate wells are shown.

### Radiation field determination

Using the 96-well plate phantom we have previously described[3], radiation was delivered using the 20 mm surface applicator. GafChromic EBT-3 film (International Specialty Products, Wayne, NJ) was used to determine the relative dose delivered for the intended radiation delivery pattern (Fig 1A). The film (Fig 1B) was digitized after 24h using an Epson Expression 10000 XL Flatbed scanner and analyzed using ImageJ and MATLAB (MathWorks, Natick MA) as previously described[3, 16]. Relative dose was determined by background subtraction of unexposed film and scaled to the dose at the center of the 4 target wells (Fig 1C).

### Reactive oxygen species assay

HTE cells were seeded (2.0x10^4^ cells/well) to achieve >90% confluence the following day in a flat-bottom 96-well plate. The next day, immediately before use, the ROS reactive dye, chloromethyl-H_2_DCFDA was dissolved in dimethyl sulfoxide (DMSO) to a 1 mM stock concentration. The dye was further diluted in phosphate buffered saline (PBS) to yield a final cell loading concentration of 5 μM. Cell culture media was removed from the 96-well plate and the cells were washed with PBS prior to loading. Chloromethyl-H_2_DCFDA dye was then loaded (5 μM final concentration in PBS) at 100 μL per well and incubated at 37°C for 5 minutes. After the dye was loaded the cells were then washed in PBS and maintained in 100 μL of phenol-red free complete media during treatment [17]. The 96-well plate was then differentially irradiated in groups of 4 wells (for sample replicates) using a high-throughput variable dose-rate irradiator [3]. Five minutes after radiation exposure, the cells were immediately placed on a fluorescent plate reader (SpectraMax i3, Molecular Devices, CA, USA) and total well fluorescence determined using excitation 510 nm (bandwidth 9 nM) and emission 550 nm (bandwidth 15 nM). Groups of wells were assayed based on the time from the end of irradiation to maintain a consistency. Data analysis was performed using GraphPad Prism (GraphPad Software, Inc., La Jolla, CA, USA). The average per well signal (relative fluorescence units) was graphed with the 95% confidence interval shown.

### Comet assay

HTE cells were seeded (2.0x10^3^ cells/well) and differentially irradiated 2 days later at approximately 40% confluence. Ten minutes after the completion of irradiation, media was aspirated and 50 μL of trypsin was added, cells were incubated at 37°C for 5 minutes. The 8 wells per dose point were amalgamated in PBS with 2% FBS to achieve high cell counts with >80% live cells. Cells were centrifuged at 4°C at 1600 RPM for 6 minutes and cells suspended in 50 μL PBS for cell counting with trypan blue, samples were then diluted in PBS to achieve 1.0x10^5^ cells/mL. The sample was then mixed with 37°C LM Agarose at a 1:10 ratio and 50 μL of mixed sample pipetted onto CometSlides, and the agrose was allowed to solidify at 4°C for ten minutes and immersed in Lysis solution overnight. The following day the slides were removed and placed in 4°C neutral electrophoresis buffer for 30 minutes. Electrophoresis was then performed at a voltage of 1 V/cm for 45 minutes at 4°C. Slides were then immersed in DNA precipitation solution for 30 minutes at room temperature. Following DNA precipitation, the slides were immersed in 70% ethanol for 30 minutes at room temperature before drying for 15 minutes at 37°C. To stain the samples 100 μL of diluted SYBR Green Staining Solution for 30 minutes at room temperature, the excess dye was removed and slides washed in dH_2_O before final drying for 30 minutes at 37°C. Imaging was performed using a Olympus BX41 fluorescent microscope using a FITC filter at 10X magnification and Olive tail moment of the comets calculated by CaspLab (www.casplab.com) [9]. Differences in the number of comets counted was due to differences in number of imaged cells. Data analysis was performed using GraphPad Prism. The mean Olive tail moment per cell per dose point is graphed with the 95% confidence interval of these samples utilized [10].

### γH2AX assay

HTE cells were seeded (2.0x10^4^cells/well) to achieve >90% confluence in a flat-bottom 96-well and differentially irradiated in groups of 4 wells (for sample replicates)[3]. 30 minutes post-irradiation, cells were fixed in 4% formaldehyde (methanol free) in phosphate buffered saline (PBS), pH 7.4, for 10 minutes at 37°C and then chilled on ice for one minute. Cells were washed three times with PBS and then permeabilized using 90% methanol (in PBS) for 30 minutes on ice. Cells were again washed three times with PBS and non-specific antibody binding was blocked by incubating cells in an incubation buffer for 10 minutes at room temperature. Buffer was aspirated and cells were incubated with primary antibody at a 1:400 concentration, Phospho-Histone H2A.X (Ser 139) (20E3) Rabbit mAb diluted in incubation buffer at room temperature for one hour. Cells were washed three times with antibody-free incubation buffer. Cells were next incubated with a fluorescently conjugated Alexa Fluor® 488 secondary antibody diluted 1:1000 with incubation buffer for 30 minutes at room temperature. During this incubation, cells were protected from light by wrapping in aluminum foil. Secondary antibody buffer was aspirated prior to a final 3 wash series and were maintained in PBS for γH2AX foci detection. The plate was then read on a SpectraMax i3 Multi-Mode Microplate Reader Platform with MiniMax 300 Imaging Cytometer (Molecular Devices, Sunnyvale, CA, USA) using the included SoftMax Pro software (v6.3). Using the output parameter of average integrated intensity, the cell average γH2AX fluorescence is measured. This results in γH2AX fluorescence per cell as this data is normalized by cell count [11]. The selected wells from the plate were then read and the data exported for analysis. We have utilized eight wells (of a 96 well plate) per condition which resulted in the analysis of over 1.6x10^5^ individual cells. Data analysis was performed using GraphPad Prism. The mean per well signal (relative fluorescence units) is graphed with the 95% confidence interval of these samples utilized.

## Supporting information

S1 Fig Assessment radiation effects in ^137^Cs irradiator. (a) reactive oxygen species, (b) physical DNA strand breaks, and c() DNA repair signaling in cells irradiated in a ^137^Cs irradiator.
(TIF)

S2 Fig Correlation between assays. (a) ROS and DNA DSBs, (b) ROS and γH2AX, and (c) DNA DSBs and γH2AX.
(TIF)

S1 Table Assay reagents, antibodies, and recipes. (PDF)

## References

[1] Hall EJ, Giaccia AJ. Radiobiology for the radiologist. Philadelphia: Wolters Kluwer Health/Lippincott Williams & Wilkins; 2012.

[2] Dodd B, Vetter RJ. Replacement of 137Cs irradiators with x-ray irradiators. Health physics. 2009;96(2 Suppl):S27–30. Epub 2009/01/16. doi: 10.1097/01.HP.0000334555.78657.bc . 19125053 10.1097/01.HP.0000334555.78657.bc

[3] Fowler TL, Fulkerson RK, Micka JA, Kimple RJ, Bednarz BP. A novel high-throughput irradiator for in vitro radiation sensitivity bioassays. Phys Med Biol. 2014;59(6):1459–70. Epub 2014/03/04. doi: 10.1088/0031-9155/59/6/1459 ; PubMed Central PMCID: PMCPMC4036445. 24584120 10.1088/0031-9155/59/6/1459 PMC4036445

[4] Hall WA, Bergom C, Thompson RF, Baschnagel AM, Vijayakumar S, Willers H, et al Precision Oncology and Genomically Guided Radiation Therapy: A Report From the American Society for Radiation Oncology/American Association of Physicists in Medicine/National Cancer Institute Precision Medicine Conference. Int J Radiat Oncol Biol Phys. 2017 Epub 2017/10/02. doi: 10.1016/j.ijrobp.2017.05.044 . 28964588 10.1016/j.ijrobp.2017.05.044

[5] Bhatia S, Hirsch K, Sharma J, Oweida A, Griego A, Keysar S, et al Enhancing radiosensitization in EphB4 receptor-expressing Head and Neck Squamous Cell Carcinomas. Sci Rep. 2016;6:38792 Epub 2016/12/13. doi: 10.1038/srep38792 ; PubMed Central PMCID: PMCPMC5150255. 27941840 10.1038/srep38792 PMC5150255

[6] Estrada-Bernal A, Chatterjee M, Haque SJ, Yang L, Morgan MA, Kotian S, et al MEK inhibitor GSK1120212-mediated radiosensitization of pancreatic cancer cells involves inhibition of DNA double-strand break repair pathways. Cell Cycle. 2015;14(23):3713–24. Epub 2015/10/28. doi: 10.1080/15384101.2015.1104437 ; PubMed Central PMCID: PMCPMC4825728. 26505547 10.1080/15384101.2015.1104437 PMC4825728

[7] Rothkamm K, Horn S. gamma-H2AX as protein biomarker for radiation exposure. Annali dell'Istituto superiore di sanita. 2009;45(3):265–71. Epub 2009/10/29. . 19861731

[8] Kuo LJ, Yang LX. Gamma-H2AX—a novel biomarker for DNA double-strand breaks. In vivo (Athens, Greece). 2008;22(3):305–9. Epub 2008/07/10. . 18610740

[9] Konca K, Lankoff A, Banasik A, Lisowska H, Kuszewski T, Gozdz S, et al A cross-platform public domain PC image-analysis program for the comet assay. Mutation research. 2003;534(1–2):15–20. Epub 2002/12/31. . 12504751 10.1016/s1383-5718(02)00251-6

[10] Olive PL, Banath JP, Durand RE. Heterogeneity in radiation-induced DNA damage and repair in tumor and normal cells measured using the "comet" assay. Radiation research. 1990;122(1):86–94. Epub 1990/04/01. . 2320728

[11] Fowler TL, Bailey AM, Bednarz BP, Kimple RJ. High-throughput detection of DNA double-strand breaks using image cytometry. Biotechniques. 2015;58(1):37–9. doi: 10.2144/000114248 ; PubMed Central PMCID: PMCPMC4331074. 25605579 10.2144/000114248 PMC4331074

[12] Fulkerson RK, Micka JA, DeWerd LA. Dosimetric characterization and output verification for conical brachytherapy surface applicators. Part I. Electronic brachytherapy source. Med Phys. 2014;41(2):022103 Epub 2014/02/11. doi: 10.1118/1.4862505 ; PubMed Central PMCID: PMCPMC3987645. 24506635 10.1118/1.4862505 PMC3987645

[13] Bordelon DE, Zhang J, Graboski S, Cox A, Schreiber E, Zhou OZ, et al A nanotube based electron microbeam cellular irradiator for radiobiology research. The Review of scientific instruments. 2008;79(12):125102 Epub 2009/01/07. doi: 10.1063/1.3043417 ; PubMed Central PMCID: PMCPMC2678784. 19123587 10.1063/1.3043417 PMC2678784

[14] Folkard M, Schettino G, Vojnovic B, Gilchrist S, Michette AG, Pfauntsch SJ, et al A focused ultrasoft x-ray microbeam for targeting cells individually with submicrometer accuracy. Radiation research. 2001;156(6):796–804. Epub 2001/12/14. . 11741504 10.1667/0033-7587(2001)156[0796:afuxrm]2.0.co;2

[15] Kimple RJ, Smith MA, Blitzer GC, Torres AD, Martin JA, Yang RZ, et al Enhanced radiation sensitivity in HPV-positive head and neck cancer. Cancer Res. 2013;73(15):4791–800. Epub 2013/06/12. doi: 10.1158/0008-5472.CAN-13-0587 ; PubMed Central PMCID: PMCPMC3732540. 23749640 10.1158/0008-5472.CAN-13-0587 PMC3732540

[16] McCaw TJ, Micka JA, Dewerd LA. Characterizing the marker-dye correction for Gafchromic((R)) EBT2 film: a comparison of three analysis methods. Med Phys. 2011;38(10):5771–7. Epub 2011/10/14. doi: 10.1118/1.3639997 . 21992391 10.1118/1.3639997

[17] Wan XS, Zhou Z, Kennedy AR. Adaptation of the dichlorofluorescein assay for detection of radiation-induced oxidative stress in cultured cells. Radiation research. 2003;160(6):622–30. Epub 2003/12/04. . 14640785 10.1667/3099

