# Supplementary figures and images for: Biological characterization of a novel in vitro cell irradiator
Source: PLoS One. 2017 Dec 12;12(12):e0189494. doi: 10.1371/journal.pone.0189494 (PMC5726654; doi:10.1371/journal.pone.0189494)

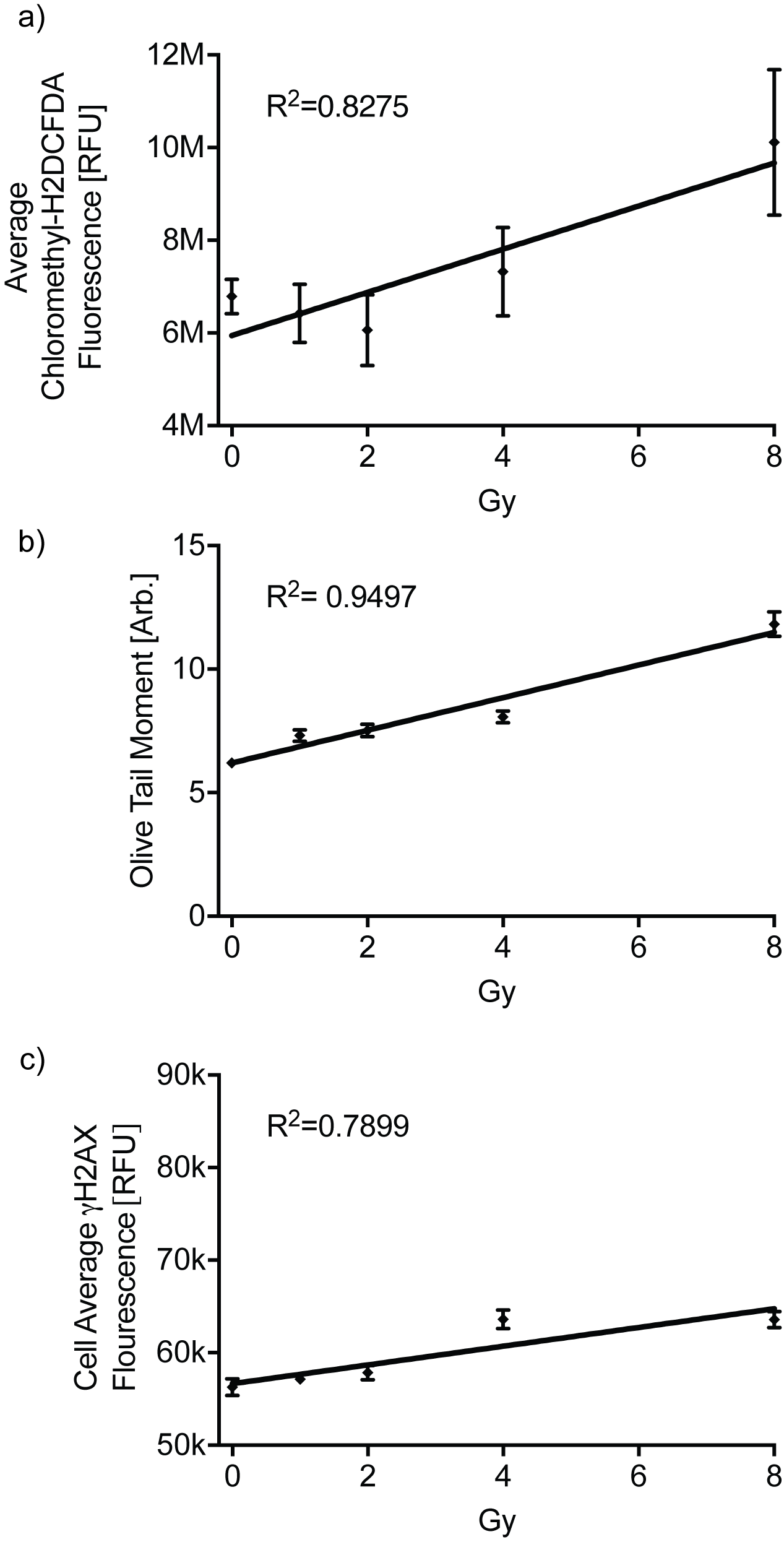

Supplement: S1 Fig — (a) reactive oxygen species, (b) physical DNA strand breaks, and c() DNA repair signaling in cells irradiated in a 137Cs irradiator. (TIF) [file pone.0189494.s001.tif]

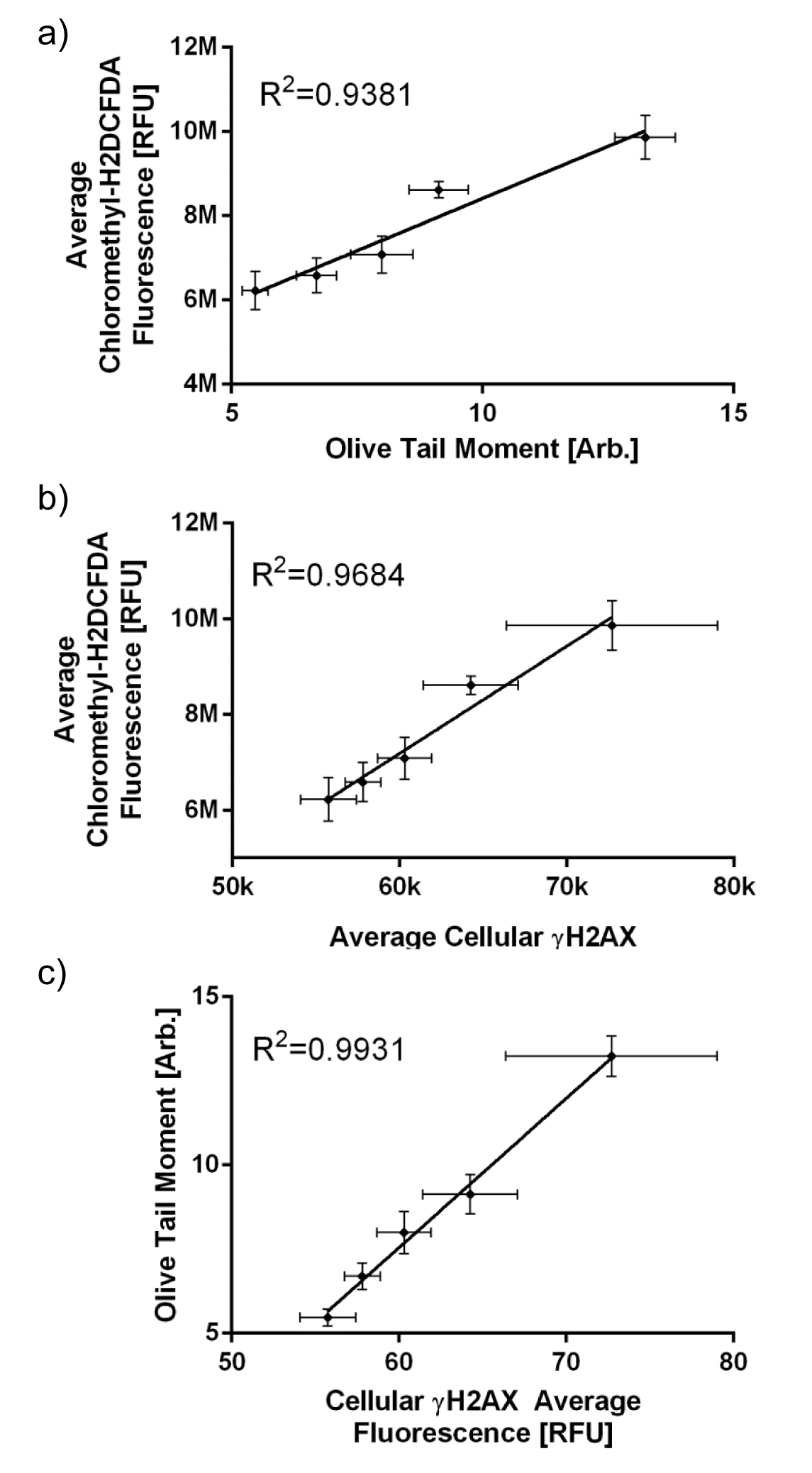

Supplement: S2 Fig — (a) ROS and DNA DSBs, (b) ROS and γH2AX, and (c) DNA DSBs and γH2AX. (TIF) [file pone.0189494.s002.tif]
