# Supplementary material for: Biological characterization of a novel in vitro cell irradiator
Source: PLoS One. 2017 Dec 12;12(12):e0189494. doi: 10.1371/journal.pone.0189494 (PMC5726654; doi:10.1371/journal.pone.0189494)
Supplement: S1 Table — (PDF) [file pone.0189494.s003.pdf]

Supplemental Table 1. Assay reagents, antibodies, and recipes.

Tissue Culture		
**Reagent**	**Manufacturer**	**Product #**
Kerotinocyte Serum Free Media Kit	Life Technologies, Grand Island, NY, USA	17005-042
Pen-Strep		
**Reactive Oxygen Species Assay**		
**Reagent**	**Manufacturer**	**Product #**
Chloromethyl-H_2DCFDA	Molecular Probes, Eugene, OR, USA	C6827
**Comet Assay**		
**Reagent**	**Manufacturer**	**Product #**
LM Agarose	Trevigen Inc., Gaithersburg, MD, USA	4250-050-02
Lysis Solution	Trevigen Inc., Gaithersburg, MD, USA	4250-050-01
CometSlides	Trevigen Inc., Gaithersburg, MD, USA	4250-200-03
SYBR Green (10,000X)	Life Technologies, Grand Island, NY, USA	S-7563
**Reagent**	**Recipe**	
TE Buffer	Tris-HCl (pH=7.5)	10 mM
	EDTA	1 mM
SYBR Green Staining Solution	SYBR Green (10,000X)	0.25 µL
	TE Buffer	7.5 mL
Neutral Electrophoresis Buffer (10X, 500 mL)	Tris Base	60.57 g
	Sodium acetate	204.12 g
	dH_2O	450 mL
	pH=9.0 (adjust pH with glacial acetic acid), adjust volume to 500 mL, filter sterilize, store at room temperature	
DNA Precipitation Solution (1X, 50 mL)	7.5M NH_4Ac	6.7 mL
	95% ETOH	43.3 mL
**γH2AX Assay**		
**Antibody**	**Manufacturer**	**Product #**
Phospho-Histone H2A.X (Ser 139) (20E3) Rabbit mAb	Cell Signaling Technology, Danvers, MA, USA	9718
Alexa Fluor® 488 secondary anti-rabbit IgG	Cell Signaling Technology, Danvers, MA, USA	4412
**Reagent**	**Recipe**	
Incubation Buffer	BSA	1 g
	PBS (pH=7.4)	200 mL
